# 

**Published:** 1876-10

**Authors:** 


### Books and Pamphlets Received.

Cyclopædia of the Practice of Medicine, Edited by Dr. H. Von Zieinssen. Vol. VI. Diseases of the Circulatory Organs; together with chapters on Whooping Cough, Diseases of the Lips and Cavity of the Mouth, and diseases of the Soft Palate. New York: Wm. Wood & Co., 1876.

A Treatise on the Theory and Practice of Medicine. By John Syer Bristow, M. D., Lond , F. R. C. P. Edited with Notes by James H. Hutchinson, M. D. Philadelphia: Henry C. Lea, 1876. Buffalo: T. Butler & Son.

A Practical Treatise on the Diseases, Injuries and Malformations of the Urinary Bladder, the Prostrate Gland and the Urethra. By Samuel D. Gross, M. D., LL. D , D. C. L. Oxon. Third edition, revised and edited by Samuel W. Gross, A. M., M. D. Philadelphia: Henry C. Lea, 1876. Buffalo: T. Butler & Son.

Epitome of Skin Diseases, with Formulæ, for Students and Practioners. By Tilbury Fox, M. D., F. R. C. P., and T. C. Fox, B. A. (Cantab.), M. R. C. S. Philadelphia: Henry C. Lea, 1876. Buffalo: T. Butler & Son.

A Century of American Medicine, 1776–1876. By Edward H. Clarke, M. D., Henry J. Bigelow, M. D., Samuel D. Gross, M., D., LL. D., etc., T. Gaillard Thomas, M. D., and J. S. Billings, M. D. Philadelphia: Henry C. Lea, 1876. Buffalo: T. Butler & Son.

A Contribution to the Treatment of Uterine Versions and Flexions. By Ephriam Cutter, A. M., M. D. Second Edition. Boston: James Campbell, 1876.

Compendium of Histology. Twenty-Four Lectures. By Heinrich Frey. Translated from the German by Geo. R. Cutter, M. D. New York: G. P. Putnams' Sons, 1876. Buffalo: Peter Paul & Bro.

Studies, Chiefly Clinical, in the Non-Emetic use of Ipecuanha: With a Contribution to the Therapeusis of Cholera. By Alfred A. Woodhull, M. D. Philadelphia: J. B. Lippincott & Co., 1876. Buffalo: Peter Paul & Bro.

### Contents of October Number.

### ORIGINAL.

THE BUFFALO

**Medical and Surgical Journal**

PUBLISHED MONTHLY,

Containing Original Articles, Reports of Medical Societies and Hospitals, Editorial, Reviews, Correspondence, Army News, etc., etc; including the usual variety of Medical Periodical Publications. Specimen copies sent on application.

Address Buffalo Medical & Surgical Journal, Buffalo, N. Y.

TERMS OF SUBSCRIPTION, $3.00 PER YEAR, IN ADVANCE.

